# Phase I study of pegylated liposomal doxorubicin and the multidrug-resistance modulator, valspodar

**DOI:** 10.1038/sj.bjc.6602653

**Published:** 2005-06-07

**Authors:** P M Fracasso, K A Blum, M K Ma, B R Tan, L P Wright, S A Goodner, C L Fears, W Hou, M A Arquette, J Picus, A Denes, J E Mortimer, L Ratner, S P Ivy, H L McLeod

**Affiliations:** 1 Alvin J Siteman Cancer Center and the Department of Medicine, Washington University School of Medicine, St Louis, MO 63110, USA; 2 Cancer Therapy Evaluation Program, Division of Cancer Treatment and Diagnosis, National Cancer Institute, Bethesda, MD, USA

**Keywords:** doxil, multidrug resistance, pegylated liposomal doxorubicin, PSC 833, valspodar

## Abstract

Valspodar, a P-glycoprotein modulator, affects pharmacokinetics of doxorubicin when administered in combination, resulting in doxorubicin dose reduction. In animal models, valspodar has minimal interaction with pegylated liposomal doxorubicin (PEG-LD). To determine any pharmacokinetic interaction in humans, we designed a study to determine maximum tolerated dose, dose-limiting toxicity (DLT), and pharmacokinetics of total doxorubicin, in PEG-LD and valspodar combination therapy in patients with advanced malignancies. Patients received PEG-LD 20–25 mg m^−2^ intravenously over 1 h for cycle one. In subsequent 2-week cycles, valspodar was administered as 72 h continuous intravenous infusion with PEG-LD beginning at 8 mg m^−2^ and escalated in an accelerated titration design to 25 mg m^−2^. Pharmacokinetic data were collected with and without valspodar. A total of 14 patients completed at least two cycles of therapy. No DLTs were observed in six patients treated at the highest level of PEG-LD 25 mg m^−2^. The most common toxicities were fatigue, nausea, vomiting, mucositis, palmar plantar erythrodysesthesia, diarrhoea, and ataxia. Partial responses were observed in patients with breast and ovarian carcinoma. The mean (range) total doxorubicin clearance decreased from 27 (10–73) ml h^−1^ m^−2^ in cycle 1 to 18 (3–37) ml h^−1^ m^−2^ with the addition of valspodar in cycle 2 (*P*=0.009). Treatment with PEG-LD 25 mg m^−2^ in combination with valspodar results in a moderate prolongation of total doxorubicin clearance and half-life but did not increase the toxicity of this agent.

Resistance to chemotherapeutic agents is an obstacle to the successful treatment of malignancies. Resistance has been attributed, in part, to the expression of the *MDR1* gene and its protein product, P-glycoprotein (Gottesman *et al*, 2002). P-glycoprotein, a member of the ATP-binding cassette superfamily of transmembrane transporters, prevents the intracellular accumulation of many natural product-derived cytotoxic agents (Gottesman *et al*, 2002). As a result, targeted inhibition of P-glycoprotein by agents administered in combination with chemotherapeutic agents has been attempted (Ford *et al*, 1996; Sikic, 1999; Leonard *et al*, 2003). One such agent, valspodar (PSC 833), a cyclosporine D analogue, reverses P-glycoprotein-mediated resistance *in vitro* at concentrations of 1000 ng ml^−1^ (Twentyman and Bleehen, 1991). In phase 1 studies, combinations of valspodar with single agent etoposide, doxorubicin, paclitaxel, or vinblastine are feasible with toxicities consisting of reversible cerebellar ataxia, myelosuppression, and hyperbilirubinemia (Boote *et al*, 1996; Giaccone *et al*, 1997; Fracasso *et al*, 2000; Bates *et al*, 2001, 2004; Chico *et al*, 2001; Minami *et al*, 2001). Responses and disease stabilisation have been noted in patients with carcinomas of the ovary, lung, and kidney (Fracasso *et al*, 2000; Bates *et al*, 2001; Chico *et al*, 2001).

Valspodar has been shown to decrease the clearance of etoposide, doxorubicin, paclitaxel, and vinblastine, and these interactions have necessitated dose reductions of the anticancer agents when given as single agents in combination with valspodar (Boote *et al*, 1996; Giaccone *et al*, 1997; Fracasso *et al*, 2000; Bates *et al*, 2001, 2004; Chico *et al*, 2001; Minami *et al*, 2001). Liposomal encapsulated anticancer agents may be better suited to combination therapy with valspodar therapy, as one such agent, liposomal doxorubicin, appears to have minimal pharmacokinetic interactions with valspodar in mice, in comparison to free doxorubicin (Krishna and Mayer, 1997; Krishna *et al*, 2000). Additional *in vivo* studies have demonstrated that the elimination of liposomal doxorubicin from the plasma via renal and hepatic mechanisms is minimally affected by valspodar (Krishna *et al*, 1999). These animal studies would suggest that the FDA-approved anticancer agent, pegylated liposomal doxorubicin (PEG-LD) (Doxil®) may be administered at full dose in combination with valspodar to patients with malignancies, thus allowing for equivalent anticancer drug exposure with the added benefit of P-glycoprotein inhibition and no additional toxicity.

Given the activity of PEG-LD in various malignancies, including Kaposi's sarcoma and breast and ovarian carcinoma (Northfelt *et al*, 1998; Gordon *et al*, 2000; Campos *et al*, 2001; Sparano and Winer, 2001), and its minimal interactions with valspodar in animal studies (Krishna and Mayer, 1997; Krishna *et al*, 1999, 2000), we proposed a phase I study with this combination. This study was designed to determine the maximum tolerated dose (MTD) and dose-limiting toxicity (DLT) using a one-patient-per-cohort accelerated titration design as proposed by Simon *et al* (1997). In addition, this study evaluated the toxicities of the combination of PEG-LD and valspodar and investigated the effects of valspodar on the pharmacokinetics of doxorubicin. Treatment responses were observed, but were not a primary end point.

## PATIENTS AND METHODS

### Patient eligibility

This study was initially written and approved for treatment of patients with AIDS-related Kaposi's sarcoma. However, given the decline in Kaposi's sarcoma with the use of highly active antiretroviral therapy (HAART) therapy and the known activity of PEG-LD in breast and ovarian malignancies, this protocol was amended to include patients with a histologically documented resistant or recurrent malignancies. A life expectancy >4 months, age ⩾18 years, and laboratory parameters (haemoglobin ⩾8 g dl^−1^, absolute neutrophil count ⩾1000 cells *μ*l^−1^, platelet count ⩾75 000 cells *μ*l^−1^, creatinine ⩽2.0 mg dl^−1^, SGOT ⩽2 × the institutional upper limit of normal (ULN), and bilirubin <1.5 × the institutional ULN) were required. Prior cytotoxic or radiation therapy had to be completed 4 weeks prior to enrollment. Prior chemotherapy with PEG-LD and doxorubicin was permitted. Patients with congestive heart failure or neuropathy (motor or sensory), or with a history of prior PEG-LD or cyclosporine A hypersensitivity were excluded. Any patient receiving medications that are metabolised by the cytochrome *P*450 system including calcium channel blockers, imidazole antifungal agents, anticonvulsants, or macrolide antibiotics had to discontinue these medications 48 h prior to valspodar therapy, during valspodar administration and up to 48 h after the last dose of valspodar in a cycle. The Cancer Therapy Evaluation Program (T97-0073), Division of Cancer Treatment and Diagnosis, National Cancer Institute (NCI) and the Washington University Human Studies Committee approved this protocol. All patients provided their signed informed consent prior to study entry.

### Treatment plan

This study used a treatment cycle of 14 days, similar to the treatment schedule of PEG-LD used by Northfelt *et al*, (1998) for AIDS-related Kaposi's sarcoma. In cycle 1, patients in dose levels 1 through 7 received PEG-LD (Doxil®, Ortho Biotech Products, L.P., Raritan, NJ, USA) alone at 20 mg m^−2^ intravenously over 1 h. Patients in dose levels 8 and 9 received PEG-LD at 22 and 25 mg m^−2^ during cycle 1, respectively. Beginning with cycle 2, PEG-LD was administered at a starting dose of 8 mg m^−2^ in combination with a fixed dose of valspodar and dose escalated in 2 mg m^−2^ increments (dose levels 2–8) to a final dose escalation to 24 mg m^−2^ (dose level 9). However, the protocol was amended after the treatment of patient #8 to change the final dose level to PEG-LD 25 mg m^−2^ (administered every 2 weeks). Valspodar (PSC 833, Amdray®, Novartis Pharmaceuticals Corporation, East Hanover, NJ, USA) is typically administered prior to the antineoplastic agent. However, given the long half-life of PEG-LD, valspodar was administered immediately after the PEG-LD infusion as a 1.42 mg kg^−1^ h^−1^ infusion over 2 h and then reduced to 0.42 mg kg h^−1^ (10 mg kg d^−1^) for an additional 70 h. Administered in this way, valspodar maintains levels above 1000 ng ml^−1^, the concentration necessary to reverse P-glycoprotein-mediated resistance *in vitro*, for 72 h.

Patients were not treated at all dose levels because we utilised the one-patient-per-cohort accelerated titration design (#4) as proposed by Simon *et al*, 1997) described below. Briefly, this design began with the treatment of one patient at the lowest dose level (PEG-LD 8 mg m^−2^). Intrapatient double dose escalation (i.e. 4 mg m^−2^ *vs* usual single dose increments of 2 mg m^−2^) occurred at the completion of each cycle unless the patient experienced a DLT or two instances of grade ⩾2 nonhaematologic toxicity during any cycle. Provided the patient(s) on the previous dose level had no DLT and less than two instances of grade 2 nonhaematologic toxicity during any cycle, the next patient enrolled began therapy at the new escalated dose level. Intrapatient and interpatient dose escalation in the absence of the toxicity as defined above was permitted, such that dose escalation could occur simultaneously for two (or more) patients at different dose levels. Once a DLT or two instances of grade ⩾2 nonhaematologic toxicity occurred, the accelerated escalation phase was stopped, and the design reverted to a modified Fibonacci scheme with three patients enrolled at this dose level. If zero of three patients experienced DLT, dose escalation resumed in single dose increments (2 mg m^−2^). If one of three experienced DLT, three more patients were enrolled at the same dose level. If two of three patients experienced a DLT, the previous dose level was expanded to six patients. The MTD was that dose in which no greater than one of six patients experienced a DLT.

Dose-limiting toxicity was defined during the second cycle only as grade 4 neutropenia or thrombocytopenia of >7 days duration or a failure to recover the absolute neutrophil count (ANC) to ⩾1000 cells *μ*l^−1^ or the platelet count to ⩾75 000 cells *μ*l^−1^ by day 21. Any grade 3 or 4 nonhaematologic toxicity excluding alopecia, nausea, vomiting, fever, anorexia, or mucositis were also considered dose-limiting. For valspodar, grade 3 or 4 cerebellar dysfunction, despite a prior 25% dose reduction in valspodar, was also considered a DLT.

Dose modifications and delays were permitted as follows: an ANC <1000 cells *μ*l^−1^ or a platelet count <75 000 cells *μ*l^−1^ by day 1 of the subsequent cycle necessitated a 1 week treatment delay. PEG-LD doses were decreased by one dose level if the ANC was <500 cells *μ*l^−1^ for >7 days (grade 4 neutropenia), if the patient experienced febrile neutropenia, or if the ANC failed to recover to ⩾1000 cells *μ*l^−1^ by the next cycle. In the event of grade 3 skin toxicity, palmar-plantar erythrodysesthesia (PPE), or mucositis, a 25% dose reduction occurred in the PEG-LD and treatment was delayed until the toxicity resolved to a grade 1. In the case of grade 3 cerebellar dysfunction, valspodar was reduced by 25%.

Antitumour response was evaluated after the second cycle of therapy (i.e. 4 weeks) and subsequently, after every four cycles (i.e. every 8 weeks). Responses were defined according to the RECIST criteria (Therasse *et al*, 2000) except for the patient with Kaposi's sarcoma, whose response was defined according to the criteria used by the World Health Association (WHO Handbook, 1979).

### Pharmacokinetic monitoring

During the first (PEG-LD alone) and the second (PEG-LD and valspodar) cycles, patients had blood samples obtained at sites contralateral to the infusion site and collected in heparinised tubes before PEG-LD infusion, and 1, 2, 6, 8, 24, 48, and 72 h after the start of the PEG-LD infusion. Plasma samples were stored at −70°C until analyses.

Plasma (0.5 ml) with internal standard (daunorubicin 1000 ng) was combined with 3% (v v^−1^) triton X-100 (50 *μ*l), vortexed for 10 s to disrupt the liposome, and vortex mixed with 65% (w v^−1^) 5-sulphosalicylic acid (50 *μ*l) for 10 s to extract doxorubicin (Chin *et al*, 2001). The reagent mixture (610 *μ*l) was then centrifuged at 14 000 r.p.m. for 10 min at room temperature. The extracted supernatant was removed and reconstituted in 75 *μ*l of 3 M sodium acetate and a final volume of 50 *μ*l was injected onto a column using a fluorescence detection method. Briefly, a HPLC pump LC-10AD and controller SCL-10A (Shimadzu Scientific instruments, Columbia, MD, USA) were used to deliver a mobile phase of 50 mM potassium dihydrogen phosphate, pH 3.0: acetonitrile (75 : 25, v v^−1^) with 10 mg ml^−1^ desipramine isocratically at a flow rate of 1.0 ml min^−1^ to precolumn premier C18 (120A) and a Shimadzu chromegabond® TC 18 column (4.6 × 100 mm, 5 *μ*m) (Shimadzu Scientific Instruments, Columbia, MD, USA). Desipramine was used to reduce doxorubicin adsorption to the HPLC system (Gabizon *et al*, 1993). Samples were injected with a Shimadzu SIL-10AD autosampler. Fluorescence detector RF 10AXL (Shimadzu Scientific Instruments, Columbia, MD, USA) was used to quantitate total doxorubicin concentrations in the presence of the internal standard daunorubicin, using excitation and emission wavelengths of 470 and 590 nm, respectively. Total doxorubicin concentrations were linear within the concentration range of 25–1000 ng ml^−1^. The lower limit of quantitation for this assay was 25 ng ml^−1^. Samples containing concentrations above the highest calibration standard were reanalysed after dilution with blank plasma up to a 1 : 40 dilution. In all, 20 replicates of a low (35 ng ml^−1^), intermediate (150 ng ml^−1^), and high (600 ng ml^−1^) concentration were repeated on three consecutive days to determine the accuracy and precision of the assay. Intra-assay variability was 1.9, 3.3, and 3.8% at 35, 150, and 600 ng ml^−1^, respectively. Interassay variability was within 5% across the linear range of the calibration curve. All validation samples were within 10% of the corresponding spiked concentrations.

The disposition of total doxorubicin was fit with both one-and two-compartment model with linear distribution and elimination. The parameters estimated in the linear model included apparent volume of the central compartment (V) and the intercompartmental first-order rate constants *K*_12_, *K*_21_, and *K*_10_. Pharmacokinetic parameters for each set of patient data were fit using an iterative two-stage analysis with maximum-likelihood (ML) estimation, as implemented in ADAPT II software (D'Argenio and Schumitzky, 1979), and the median for each parameter was then used as the revised initial estimates for the next ML iteration. The median for each parameter was updated until the parameter estimates for all parameters were stable (defined as no net change to the second significant digit). Total doxorubicin concentration–time data from each patient was fit individually for each cycle. All modelling of cycle 2 data included any residual total doxorubicin concentrations detectable in the pretreatment sample (time=0). Each data set was assessed for the goodness of model-curve fit by an estimate of the variance for the predicted values, correlation coefficients, and the Akaike information criterion (AIC) between different models.

Area under the concentration *vs* time curves (AUC_0 → ∞_) was calculated by integration of the simulated concentration–time data from the model estimates. Systemic clearance was calculated as *V* × *K*_10_. The alpha and beta elimination half-lives were calculated using equations generated by ADAPT II software. The differences in total doxorubicin clearance, terminal half-life, or total doxorubicin AUC between cycle 1 and 2 were assessed using Wilcoxon matched pairs test. The correlation between PEG-LD dose and AUC was assessed using the Spearman's rank test. All comparisons were considered significant at *P*<0.05.

## RESULTS

### Patients

The characteristics of the 14 patients enrolled from March 1998 until December 2001 are summarised in Table 1. Six patients had received prior therapy with doxorubicin and one patient had received prior therapy with PEG-LD. Pegylated liposomal doxorubicin and valspodar was first-line therapy for the patients with hepatoma and Kaposi's sarcoma.

### Treatment administered

All patients completed at least two cycles of therapy, with cycle 1 containing PEG-LD alone and cycle 2 containing both PEG-LD and valspodar (Table 2). A total of 69 combined PEG-LD and valspodar treatment cycles were administered, (range 1–13). As per study design, intrapatient double dose escalation was terminated after patient #2 experienced a DLT (grade 3 PPE) at PEG-LD 24 mg m^−2^. This patient with hepatoma received a total of 14 cycles after a 25% dose reduction at cycle 5 and again at cycle 10 (see Table 2). The traditional single-step dose escalation with three patients per dose level was then initiated with patient #3 receiving PEG-LD 20 mg m^−2^, the previous dose level for which patient #2 had no significant toxicities. Since no DLTs were observed at PEG-LD 20 and 22 mg m^−2^, six patients received PEG-LD 25 mg m^−2^ in combination with valspodar.

In all, 90% of the 69 cycles of combined PEG-LD and valspodar were administered on schedule. Seven cycles administered to four patients (#2, 6, 12, and 14) were delayed due to PPE. These four patients also underwent dose reductions (as noted in Table 2) following the dose delay. In these four patients, all dose delays and reductions occurred after receiving at least three cycles of PEG-LD (one cycle of PEG-LD alone and several cycles in combination with valspodar) at a dose of >20 mg m^−2^.

### Toxicity

Table 3 summarises the haematologic toxicities for all treatment cycles. Only two grade 3 haematologic toxicities were noted. Patient #12 experienced grade 3 neutropenia and PPE after four cycles of treatment with PEG-LD 25 mg m^−2^, which led to a subsequent dose delay and reduction, and patient #4 experienced grade 3 anemia requiring a red blood cell transfusion after cycle 1. In addition, patients #5, 6, 8, and 14 with grade 2 anemia received red blood cell transfusions, and patients #4, 5, 13, and 14 received erythropoietin.

Table 4 summarises the most common nonhaematologic toxicities. Grade 3 and 4 events were uncommon and consisted primarily of PPE. Patient #9 developed the only grade 4 toxicity, PPE, after receiving four cycles of PEG-LD 25 mg m^−2^. Patients #2, 6, 12, and 14 experienced grade 3 PPE after they received more than three cycles of therapy at PEG-LD at doses exceeding 20 mg m^−2^. Other grade 3 events included fatigue, infection without neutropenia (cellulitis), diarrhoea, nausea/vomiting, PEG-LD infusion reaction, and valspodar-related ataxia. The PEG-LD reaction consisted of flushing, tightness, and abdominal pain. This resolved after administration of dexamethasone and diphenhydramine and did not occur with subsequent cycles after premedication with these agents. During the valspodar infusion, nine patients developed symptoms consisting of perioral numbness and tingling, paresthesias, chest discomfort, dizziness, lightheadedness, or ataxia. These symptoms were mild (grade 1) and resolved shortly after the completion of the infusion except in patient #13 who developed grade 3 ataxia which resolved several days later. This patient was never retreated with PEG-LD and valspodar due to progressive disease.

### Response

Two of the 14 patients achieved a partial response (PR). Patient #6 who had received seven prior chemotherapy regimens for metastatic breast carcinoma achieved a PR lasting for 9 weeks. Patient #12 with ovarian carcinoma who had previously received PEG-LD achieved a partial response for 19 weeks. After 12 cycles of therapy, this patient requested that she be removed from the study, as the infusion pump required for administration of valspodar was inconvenient for her lifestyle. A patient with Kaposi's sarcoma had a near PR (49% decrease in target skin lesions). This patient had s.d. for 12 weeks and after five cycles of therapy, this patient refused additional therapy. An additional four patients with breast (two patients), hepatoma, primary peritoneal, and rectal carcinoma had stable disease (range 4–22 weeks). Patient #2 with hepatoma received 13 cycles of the combination therapy with a best response of stable disease. This patient was removed from study to undergo curative surgical resection, although, at the time of resection, this was not possible.

### Pharmacokinetics

**Figure 1:**
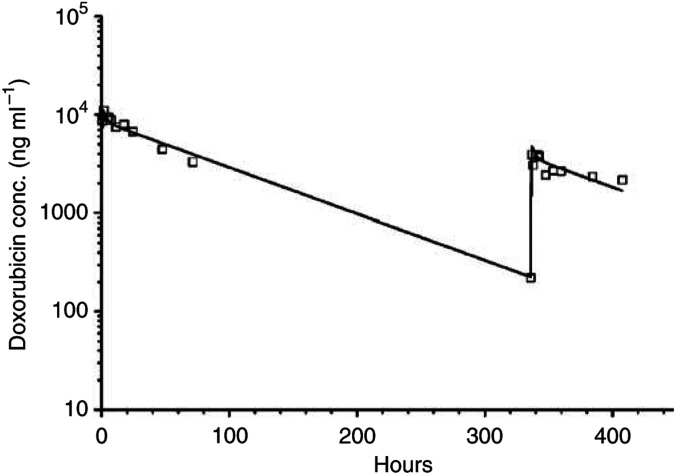
Plasma doxorubicin concentration *vs* time plot in patient 1 after receiving intravenous doxorubicin at 20 mg m^−2^ over 60 min in cycle 1. A second intravenous doxorubicin dose at 8 mg m^−2^ was started 14 days (336 h) after the first dose in combination with valspodar at a dosing rate of 1.42 mg kg^−1^ h^−1^ over 2 h, followed by 0.42 mg kg^−1^ h^−1^ over an additional 70 h. Symbols represent measured plasma doxorubicin concentrations, and the solid line represents the best fit from the maximum likelihood estimation using ADAPT II software.

All patients had total doxorubicin pharmacokinetics performed either alone and in combination with valspodar. Initial evaluation with a one-compartment model was used based on a previous study of PEG-LD disposition in children (Marina *et al*, 2002) and was able to describe total doxorubicin pharmacokinetics in the current study. However, both the AIC score and the goodness of fit were inferior to the two-compartment model (data not shown). Figure 1 depicts a best-fit line for total doxorubicin concentration–time data in patient #1 (PEG-LD 8 mg m^−2^) during cycles 1 and 2. Maximum-likelihood analysis was updated and two iterations were required to achieve stable estimates of the median parameters. The mean parameters (CV%) observed in the study include *K*_10_=0.02 h^−1^ (61%), *V*=1.48 l m^−2^ (29%), *K*_12_=0.21 (140%) h^−1^, and *K*_21_=0.64 h^−1^ (69%). Total doxorubicin pharmacokinetic parameters by patient are listed in Table 5. The interindividual variability in total doxorubicin clearance was seven-fold (10–73 mL h m^−2^) in cycle 1 and over 12-fold (3–37 ml h^−1^ m^−2^) in cycle 2. The correlation between dose and total doxorubicin clearance was weak (*R*_s_=0.06). The mean (range) total doxorubicin clearance decreased from 27 (10–73) ml h^−1^ m^−2^ in cycle 1 to 18 (3–37) ml h^−1^ m^−2^ with the addition of valspodar in cycle 2 (*P*=0.009). A three-fold range in *V* was observed in cycle 1 and there was no significant difference in V between cycles 1 and 2 (*P*=0.43). The estimated mean (range) terminal elimination half-life was 89 h (40–336 h) in cycle 2, much longer than estimated in cycle 1 (*P*=0.001). The AUC was also slightly higher in cycle 2 (mean 1689 *μ*g h ml^−1^, range 492–6257 *μ*g h ml^−1^) than in cycle 1 (mean 1087 *μ*g h ml^−1^, range 343–2408 *μ*g h ml^−1^; *P*=0.04). In this small set of patients, neither toxicity nor response was clearly related to total doxorubicin AUC or clearance.

## DISCUSSION

Clinical trials in solid tumour malignancies using the P-glycoprotein modulator, valspodar, combined with single agent etoposide, doxorubicin, paclitaxel, or vinblastine have required dose reductions of the antineoplastic agents (Boote *et al*, 1996; Giaccone *et al*, 1997; Fracasso *et al*, 2000; Bates *et al*, 2001, 2004; Chico *et al*, 2001; Minami *et al*, 2001). These dose reductions are necessary in order to reduce toxicity due to the decreased clearance and increased AUC of these agents when given in combination with valspodar. A theoretical concern has been that these dose reductions may limit drug exposure to the tumour, thereby decreasing tumour cell kill. Combining valspodar with PEG-LD may overcome this limitation as animal studies have revealed no pharmacokinetic interactions between these drugs (Krishna and Mayer, 1997; Krishna *et al*, 1999, 2000). In addition, since liposomes reduce the exposure of entrapped anticancer agent to susceptible tissues while increasing the drug delivery to tumours, PEG-LD may provide maximal drug intensity with minimal toxicity (Krishna and Mayer, 1999). In this study, we demonstrated that valspodar can be given safely with acceptable toxicity at PEG-LD 25 mg m^−2^ every 2 weeks. There was no DLT at this dose level after cycle 2 (PEG-LD and valspodar). No further dose escalation was attempted as we reasoned that PEG-LD 25 mg m^−2^ every 2 weeks equaled the cumulative dose of 50 mg m^−2^ every 4 weeks, the approved dose of PEG-LD used in the treatment of breast and ovarian carcinoma. More importantly, most patients required dose reductions due to PPE or neutropenia after multiple cycles of this combination at this dose level as well as at lower dose levels. When administered on this schedule, the pharmacokinetics of total doxorubicin in combination with valspodar was moderately affected.

The pharmacokinetic data collected in this study suggested that valspodar does moderately increase plasma levels and decrease the clearance of total doxorubicin. Nevertheless, this reduced clearance seemed to have little effect on the toxicity profile of PEG-LD when given at the 25 mg m^−2^ dose. Large interpatient variability (seven-fold in cycle 1 and 12-fold in cycle 2) in total doxorubicin clearance was evident, which was consistent with that found in other adult studies (4–40 fold) (Gabizon *et al*, 1991, 1994; Amantea *et al*, 1997). One patient (#6) had a dramatic decrease in clearance in cycle 2 with resulting high AUC. The patient was on no other known inhibitors of cytochrome *P*450 and the mechanism for this observation is not clear. The long half-life is not surprising for a pegylated liposomal drug and increased doxorubicin release was previously demonstrated in preclinical and clinical studies with this composition (Papahadjopoulos *et al*, 1991; Gabizon, 1992; Huang *et al*, 1994; Amantea *et al*, 1997). During the second cycle in which PEG-LD and valspodar are combined, the total doxorubicin half-life is even longer. This apparent increase in total doxorubicin half-life during second cycle could be in part an artefact of ‘carry-over’ from cycle 1 rather than purely a pharmacokinetic interaction between valspodar and doxorubicin. However, three patients (Patients #7, 9, and 11) who had no residual plasma doxorubicin prior to their second cycle, all consistently had 50% reduction in total doxorubicin clearance in cycle 2 supporting the conclusion that this finding was not an estimation artefact from the residual drug. Therefore, the reduction in total doxorubicin clearance with the addition of valspodar in cycle 2 was likely a result of a pharmacokinetic interaction, similar to that seen in other models where the clearance of single agent cytotoxic agents such as etoposide, doxorubicin, paclitaxel, and vinblastine was significantly reduced by valspodar administration (Boote *et al*, 1996; Giaccone *et al*, 1997; Fracasso *et al*, 2000; Bates *et al*, 2001, 2004; Chico *et al*, 2001; Minami *et al*, 2001). An influence of repeated administrations of PEG-LD on this interaction could not be assessed, as all patients received valspodar in combination with PEG-LD after cycle 1.

Despite this effect on total doxorubicin clearance, valspodar did not seem to significantly alter the toxicities of PEG-LD. Patients with the highest total doxorubicin AUC (>2000 *μ*g h ml^−1^) after cycle 2 of therapy had similar toxicities to other patients. Myelosuppression, an uncommon toxicity with PEG-LD, did not appear to be exacerbated by valspodar. Only one patient experienced grade 3 neutropenia and no episodes of neutropenic fever occurred. As would be expected, dermatologic and gastrointestinal events were more commonly observed. PPE occurred more often in patients receiving greater than three cycles of PEG-LD and valspodar and often led to dose reductions. As is the case with single agent studies with PEG-LD, this toxicity is more common after cumulative doses of PEG-LD. Nausea and vomiting occurred more frequently in patients after the first cycle when the combination therapy was administered as opposed to the first cycle when only PEG-LD was given as a single agent. This is a known toxicity with valspodar, although it is possible that the combination exacerbated this toxicity. Nevertheless, the majority of these events were grade 1–2 and controllable with antiemetic therapy.

In conclusion, the combination of PEG-LD 25 mg m^−2^ given with a 72 h infusion of valspodar can be administered every 2 weeks with acceptable toxicity. The pharmacokinetic interaction is moderate. Responses were noted in patients with breast and ovarian carcinoma at this dose of PEG-LD. However, the 72 h infusion makes this an inconvenient regimen. In addition, there are more potent third-generation, highly specific, P-glycoprotein modulators in clinical trials. One such trial in Europe, a phase 2 study with docetaxel and the oral agent, zosuquidar, in women with metastatic breast carcinoma, is ongoing. Should this be a positive study, additional studies with PEG-LD and this agent should be considered in the treatment of breast carcinoma.

## Figures and Tables

**Table 1:** Patient characteristics

**Characteristics**		**No. of patients**
		14
*Age*		
Mean	54	
Range	34–80	
		
*Sex*		
Male : female		2:12
		
*ECOG performance status*		
0		3
1		9
2		2
		
*Diagnosis*		
Breast		4
Colorectal		3
Sarcoma (leiomyosarcoma, Kaposi's sarcoma)		2
Other (hepatoma, ovarian, head and neck, primary peritoneal, renal cell carcinoma)		5
		
*Prior therapy*		
Chemotherapy		12
Median # of regimens (range)	3 (0–7)	
Prior doxorubicin		6
Mean dose (range) in mg m^−2^	337 (100–555)	
Prior PEG-LD		1
Dose (mg m^−2^)	279	
Radiation therapy		7

PEG-LD=pegylated liposomal doxorubicin.

**Table 2:** Summary of all patients and treatment administered

	**Cycle # and dose of PEG-LD (mg m^−2^)**													
**Patient #**	**1^a^**	**2**	**3**	**4**	**5**	**6**	**7**	**8**	**9**	**10**	**11**	**12**	**13**	**14**
1	20	8	12	16	20									
2	20	16	20	24	18	18	18	18	18	13.5	13.5	13.5	13.5	13.5
3	20	20	20	20										
4	20	20												
5	20	20												
6	20	22	22	16.5	16.5	16.5	16.5	12.4	12.4	12.4	12.4	12.4		
7	22	22	22	22										
8	22	22	22	22										
9	25	25	25	25										
10	25	25												
11	25	25												
12	25	25	25	25	18.75	18.75	18.75	18.75	18.75	18.75	18.75	18.75		
13	25	25												
14	25	25	25	25	25	18.75	18.75	18.75	18.75	18.75	14.06	14.06	14.06	14.06

a All patients received PEG-LD (pegylated liposomal doxorubicin) alone for the cycle 1 and PEG-LD in combination with valspodar for all other cycles.

**Table 3:** Hematologic toxicities

**Patient #**	**PEG-LD (mg m^−2^)**	**No. of PEG-LD and valspodar cycles^a^**	**ANC nadir mean (range) (cells *μ*l^−1^)**	**Platelet nadir mean (range) (10^3^ *μ*l^−1^)**	**Haemoglobin mean (range) (g dl^−1^)**
1–14	20–25	0	3.803 (1.994–7.571)	271 (161–424)	11.2 (6.7–13.8)
1	8	4^b^	2.631 (1.613–3.602)	220 (195–229)	13.5 (12.8–14.1)
2	16	13^c^	3.106 (2.109–5.083)	399 (295–529)	12.1 (10.8–12.8)
3–5	20	5	3.730 (2.275–6.255)	311 (66–501)	10.2 (8.3–12.5)
6–8	22	17^d^	2.826 (1.386–5.039)	265 (113–464)	11.0 (8.6–13.8)
9–14	25	30^e^	2.298 (0.936–7.056)	298 (137–477)	11.6 (8.7–14.0)

a Total number of cycles does not include cycle 1 (PEG-LD alone).

b Patient #1 had dose escalations of 12, 16, and 20 mg m^−2^.

c Patient #2 received dose escalations of 20 and 24 mg m^−2^ before dose reductions of 18 mg m^−2^ for five cycles and 13.5 mg m^−2^ for five cycles.

d Patient #6 was dose reduced to 16.5 mg m^−2^ for four cycles and 12.4 mg m^−2^ for five cycles.

e Patient #12 was dose reduced to 18.75 mg m^−2^ for five cycles and 14.06 mg m^−2^ for four cycles. Patient #14 was dose reduced to 18.75 mg m^−2^ for eight cycles.

PEG-LD=pegylated liposomal doxorubicin.

**Table 4:** Most common nonhaematologic toxicities

	**Cycle 1^a^**					**All other cycles^b^**				
	**Grade**					**Grade**				
**Toxicity**	**0**	**1**	**2**	**3**	**4**	**0**	**1**	**2**	**3**	**4**
*Constitutional*										
Fatigue/malaise	10	2	1	1	—	13	1	—	—	—
										
*Dermatology*										
PPE	14	—	—	—	—	9	—	—	4	1
										
*Gastrointestinal*										
Diarrhoea	10	2	2	—	—	10	2	1	1	—
Nausea/vomiting	9	4	1	—	—	4	6	3	1	—
Stomatitis/dysphagia	14	—	—	—	—	7	5	2	—	—
										
*Infection w/o neutropenia*	10	1	2	1	—	12	1	1	—	—
										
*Other*										
PEG-LD infusion reaction	13	—	—	1	—	14	—	—	—	—
Valspodar-related events	—	—	—	—	—	5	8	—	1	—

a PEG-LD (pegylated liposomal doxorubicin) alone.

b PEG-LD given in combination with valspodar.

**Table 5:** Patient pharmacokinetic parameters

	**PEG-LD (mg m^−2^) Cycle 1**	**Vd (l m^−2^) Cycle 1**	**Clearance (ml h^−1^ m^−2^) Cycle 1**	**Terminal half-life (h) Cycle 1**	**AUC _0 → ∞_ (*μ*g h ml^−1^) Cycle 1**
**Patient #**	**Cycle 2**	**Cycle 2**	**Cycle 2**	**Cycle 2**	**Cycle 2**
1	20	1.4	32	44	622
	8	2.3	17	101	492
					
2	20	0.9	12	63	1693
	16	0.9	13	88	1301
					
3	20	1.6	25	44	789
	20	1.8	19	65	1041
					
4	20	1.4	18	55	1115
	20	1.6	17	65	1175
					
5	20	1.2	17	48	1165
	20	1.2	19	52	1082
					
6	20	1.0	26	50	784
	22	1.5	3	336	6257
					
7	22	0.7	38	40	581
	22	1.4	20	48	1090
					
8	22	1.8	38	43	579
	22	2.0	32	45	690
					
9	25	2.0	22	63	1122
	25	1.7	12	102	2470
					
10	25	1.9	29	48	870
	25	1.7	20	60	1299
					
11	25	2.3	73	34	343
	25	1.4	37	40	684
					
12	25	1.1	11	73	2279
	25	0.9	11	83	2348
					
13	25	1.4	29	48	869
	25	1.8	27	55	945
					
14	25	1.1	10	99	2408
	25	1.1	10	101	2767
					
Mean Cycle 1		1.4	27	54	1087
CV%		32	60	31	58
					
Mean Cycle 2		1.5	18	89	1689
CV%		27	49	84	88

PEG-LD=pegylated liposomal doxorubicin.
